# A Rat Tail Cyclic Compression Model for Studying Intervertebral Disc Mechanobiology

**DOI:** 10.1002/jsp2.70226

**Published:** 2026-11-13

**Authors:** Kaixuan Jiang, Wei Cui, Yin Zhuang, Daoguang Tian, Wei Peng, Shujun Zhang

**Affiliations:** ^1^ Wuxi Ninth People's Hospital Affiliated to Soochow University Wuxi Jiangsu China; ^2^ Wuxi Orthopedic Institute Wuxi Jiangsu China; ^3^ Minimally Invasive Orthopedic Institute of Soochow University Wuxi Jiangsu China

**Keywords:** disc deformation, intervertebral disc, mechanical loading, mechanobiology, rat tail model

## Abstract

**Background:**

Intervertebral disc (IVD) responses vary with loading magnitude, frequency, and duration. Existing loading systems are often mechanically complex and costly, while device input alone may not represent disc deformation. We developed a five‐channel motor‐driven system to relate graded cyclic displacement to mechanical and tissue responses.

**Methods:**

Twenty 11‐week‐old male Sprague–Dawley rats were assigned to sham, low (0.4 mm), moderate (0.8 mm), and high (1.2 mm) compression groups (*n* = 5 per group). Eight additional rats underwent characterization of peak compressive force, disc height deformation, wedge angulation, and sagittal plane anterior–posterior (A–P) translation. Outcomes included imaging, histology, cartilaginous endplate morphometry, and immunohistochemistry.

**Results:**

Displacements of 0.4, 0.8, and 1.2 mm generated peak compressive forces of 3.94 ± 0.46, 8.69 ± 1.15, and 14.54 ± 1.31 N and radiographic disc height deformations of 13.00% ± 4.05%, 31.11% ± 8.38%, and 58.08% ± 10.27%, respectively. Wedge angle and sagittal plane A–P centroid offset did not differ significantly across the four displacement states (*p* > 0.05). After 4 weeks of cyclic loading, the low compression group resembled the sham group. Moderate compression preserved disc structure and increased matrix marker positive areas. High compression caused disc narrowing, T2 signal loss, nucleus pulposus disruption, increased MMP‐13 positive area, reduced cartilaginous endplate dimensions, and more frequent and severe endplate abnormalities.

**Conclusions:**

Prescribed displacement produced graded mechanical and tissue responses, with axial disc height reduction as the predominant group level radiographic deformation. This simple, low‐cost system provides an amplitude tunable platform for intervertebral disc mechanobiology research.

## 1 Introduction

Low back pain (LBP) remains one of the leading causes of disability worldwide and imposes a substantial socioeconomic burden [1], with approximately 40% of chronic LBP cases attributed to intervertebral disc degeneration (IVDD) [2]. Although aging, genetics, and inflammation contribute to IVDD progression, mechanical loading is increasingly recognized as a critical regulator of disc homeostasis and degeneration [3, 4]. The intervertebral disc is continuously exposed to complex mechanical stimuli, including compression, tension, hydrostatic pressure, fluid flow, and osmotic stress [5]. These mechanical signals regulate cellular behavior and extracellular matrix turnover through mechanotransduction pathways [3, 6]. Physiological loading is required to maintain tissue homeostasis, whereas abnormal loading may initiate degenerative remodeling and accelerate IVDD progression [6, 7]. This distinction has motivated the development of complementary animal models that induce IVDD either through direct structural injury or through controlled alterations in the mechanical environment.

Several animal models have been developed to investigate the pathogenesis of IVDD and the biological mechanisms underlying disc degeneration. Needle puncture models are widely used because they rapidly induce disc degeneration; however, they primarily represent acute structural injury rather than mechanically driven degeneration [6, 8]. In contrast, in vivo caudal loading models maintain an intact motion segment and allow the effects of controlled mechanical exposure to be studied. Static compression models can cause annular disorganization, loss of cellularity, suppression of collagen II and aggrecan, and activation of matrix metalloproteinases [9, 10, 11]; however, continuous compression does not adequately replicate the dynamic mechanical environment encountered during daily activities and may introduce secondary effects related to prolonged immobilization [12]. Dynamic loading models more closely mimic physiological mechanical conditions and have demonstrated that disc responses vary according to loading magnitude, frequency, and duration. Ching et al. reported frequency‐dependent changes in disc height and composition [13, 14], whereas Wuertz et al. observed matrix‐promoting remodeling after shorter daily exposure and mild degenerative changes with prolonged exposure [15]. Excessive dynamic loading has also been associated with region‐dependent aggrecan degradation [16]. These studies indicate that an appropriate loading promotes matrix homeostasis and excessive loading induces degenerative changes.

Despite increasing recognition of the importance of mechanical loading in disc biology, important questions remain unresolved. Most established dynamic compression models use vertebral pins or external rings connected to Ilizarov frames, pneumatic actuators, or other force‐controlled systems. Loading magnitude is commonly reported as external force or nominal compressive stress calculated from force and estimated disc area. These measures describe the apparatus input but do not directly quantify intradiscal pressure or the deformation sustained by the disc. A practical model that relates controlled device motion to measured disc deformation and later tissue responses would therefore complement existing force‐controlled systems.

We developed a simple five‐channel rat tail system in which actuator motion is transmitted to Kirschner wires fixed in adjacent vertebral bodies. This study investigated the relationship among prescribed K‐wire displacement, peak compressive force measured at the fixation assembly, radiographically measured disc deformation, and subsequent structural and biological responses. We hypothesized that graded displacement would produce increasing peak compressive force and disc deformation, with moderate compression maintaining disc structure and matrix homeostasis and high compression inducing structural degeneration and catabolic remodeling.

## 2 Materials and Methods

### 2.1 Study Design and Reporting

This exploratory in vivo study was reported in accordance with the ARRIVE 2.0 principles. The individual rat was considered the experimental unit, and Co8–Co9 was the analyzed motion segment. The experimental factor was K‐wire displacement amplitude, and the prespecified outcomes were longitudinal DHI, endpoint MRI grade, ORS histological score, cartilaginous endplate morphology, and immunohistochemical expression of matrix and catabolic markers.

The study consisted of two experimental components. Twenty rats were included in the 4‐week cyclic loading experiment and were allocated to four groups of five animals. A separate cohort of eight additional rats was used to characterize the peak compressive force and motion segment deformation, including wedge angulation and sagittal plane anterior–posterior (A–P) translation, during the applied loading. These eight rats were not included in the four experimental groups or in the 4‐week imaging, histological, or immunohistochemical outcome analyses.

Animals were assigned to the four experimental groups before surgery; however, the specific sequence‐generation procedure was not documented. Imaging measurements, histological scoring, and immunohistochemical quantification were performed by investigators blinded to group allocation. Because this was an exploratory model‐development study, no formal a priori sample‐size calculation was performed. The sample size was set at five animals per group to match the five‐channel capacity of the loading device.

### 2.2 Experimental Animals

A total of 28 11‐week‐old male Sprague–Dawley rats (300–350 g) were obtained and acclimatized for 1 week. At 12 weeks of age, 20 animals were assigned to four experimental groups (sham, low, moderate, and high compression; *n* = 5 per group) for the 4‐week experiment. The remaining eight rats formed a separate characterization cohort and were used to measure peak compressive force, radiographic disc height deformation, wedge angulation, and sagittal plane A–P translation.

12‐week‐old rats are commonly regarded as skeletally mature in rat‐tail IVD studies [17]; therefore, this age was selected to minimize growth‐related changes in caudal vertebral dimensions and K‐wire spacing during the 4‐week experiment. Only male rats were included to limit sex‐associated heterogeneity in this initial model development study. Because sex can influence IVD structure and responses to injury or mechanical stimulation, the findings require confirmation in female animals [18, 19]. No animals were excluded from the study. All implanted animals completed the experimental protocol and were included in the final analysis. All procedures were approved by the Ethics Committee of Wuxi Ninth People's Hospital Affiliated to Soochow University (KS2026‐024DW‐01) and adhered to institutional animal care guidelines.

### 2.3 Surgical Procedure

Under isoflurane anesthesia (induction 5%, maintenance 2%–3% in 100% O_2_), Kirschner wires (1.2 mm diameter, 5 cm length) were percutaneously inserted into Co8–Co9 vertebral bodies using a handheld micro‐drill (15 000 rpm) and a custom alignment jig to ensure precise parallel positioning (Figure 1A). Anteroposterior and lateral radiographs were used to verify the position and orientation of the Kirschner wires relative to the target motion segment (Figure 1B,C). Kirschner wires were then placed into the fixation slots, and rats were secured in a custom holder permitting forelimb and head movement while preventing gross body displacement (Figure 1D). The imposed device motion was compressive. Because one wire was used in each vertebral body, eccentric placement, wire bending, or segmental buckling could add bending or shear at the motion segment. Pin sites were inspected daily for erythema, swelling, discharge, wire loosening, impaired tail movement, and skin injury. Routine care consisted of cleaning with sterile saline followed by iodine. Persistent discharge, abscess formation, wire loosening, or systemic signs would have prompted trained personnel assessment and withdrawal if humane endpoint criteria were met. All implanted animals completed the protocol.

**FIGURE 1:**
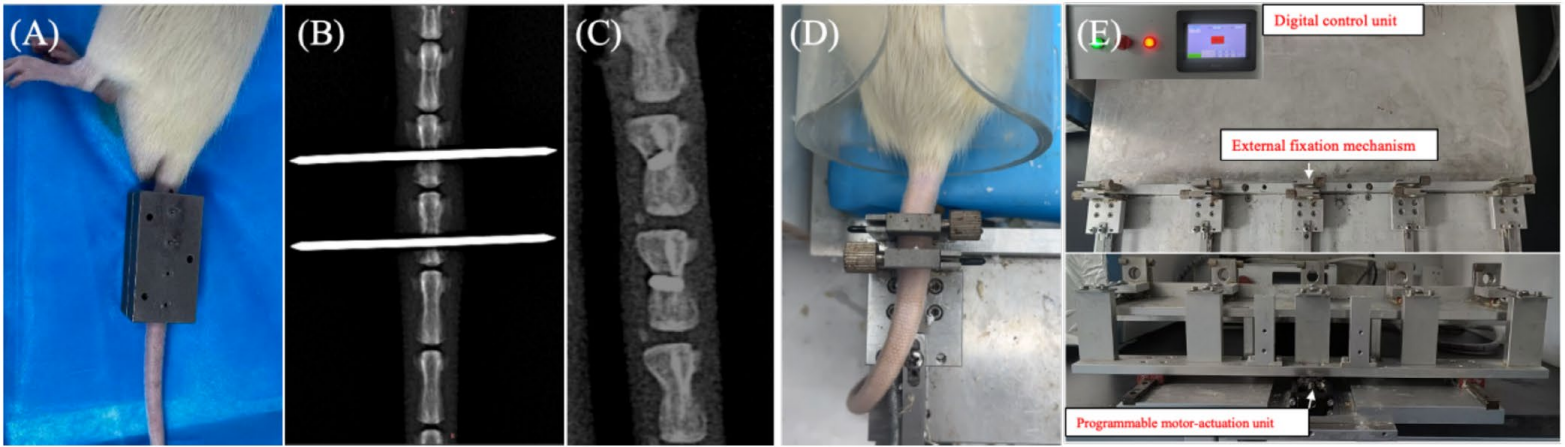
Surgical implantation and dynamic compression system. (A) Select the rat coccygeal Co8‐Co9 segment and vertically insert two Kirschner wires using a guide block. (B, C) Anteroposterior and lateral radiographs confirm Kirschner‐wire position relative to the target Co8–Co9 motion segment. (D) Connect the Kirschner wires to the device and start operation; (E) The custom dynamic compression device including the digital control unit, external fixation mechanism, and programmable motor‐actuation unit.

### 2.4 Dynamic Compression Protocol

A five‐channel motor‐driven compression device was used to deliver cyclic displacement to the Kirschner wires. The digital controller allowed adjustment of frequency and actuator output (Figure 1E). Sham animals underwent the same K‐wire implantation but were not placed in the loading device during the daily loading sessions and received no cyclic K‐wire displacement. They were allowed normal cage activity throughout the 4‐week experimental period.

The percentage value displayed by the controller represented actuator output amplitude rather than disc deformation. Mechanical calibration demonstrated that a controller setting of 1% generated approximately 0.13 mm of Kirschner wire displacement. Longitudinal changes in disc height during the 4‐week experiment were evaluated separately as DHI relative to baseline (%), calculated for each animal as (DHI at each follow‐up/DHI at baseline) × 100%. The experimental amplitudes of 0.4, 0.8, and 1.2 mm were therefore defined as prescribed K‐wire displacement rather than applied force or disc strain. These equally spaced settings covered a threefold range of device input. For readability, the corresponding groups are termed low compression, moderate compression, and high compression. These names refer to the ascending displacement settings used in this device and do not denote measured force, stress, physiological loading, or a constant percentage of disc deformation.

A loading frequency of 0.5 Hz was selected based on frequency dependent responses reported in previous in vivo rat tail studies, including greater nucleus pulposus proteoglycan content at this frequency [14]. A daily loading duration of 2 h was selected as a short exposure informed by prior protocols using a single 2 h loading session [20] and repeated loading for 1.5 h/day [15]. Thus, the combined regimen of 0.5 Hz for 2 h/day was used as a reproducible experimental setting rather than as a direct replication of any single previous study. Cyclic compression was applied at amplitudes of 0.4, 0.8, and 1.2 mm at a frequency of 0.5 Hz for 2 h/day over a period of 4 weeks. These parameters were used to provide a reproducible cyclic loading regimen and were not intended to simulate a specific rat activity or human physiological condition. The loading procedure and operation of the compression device are shown in Video S1.

In a separate cohort of eight additional instrumented rats, compressive force at peak displacement was measured in series with the loading path at the K‐wire fixation assembly using a digital force gauge aligned with the loading direction. Each rat was tested at prescribed K‐wire displacements of 0.4, 0.8, and 1.2 mm. These measurements represent the initial compressive force transmitted through the fixation assembly at peak displacement; they do not represent intradiscal force, stress, or pressure.

### 2.5 Radiographic Assessment and Motion Segment Deformation

Radiographic examinations were performed at baseline before surgery and subsequently at weeks 2, 3, and 4 after initiation of dynamic compression using a standard digital X‐ray system. During image acquisition, the rat tail was gently extended in a neutral position without rotation, and the target disc space was aligned perpendicular to the X‐ray beam.

Intervertebral disc height was quantified using the disc height index (DHI) [21], which was calculated as the sum of the anterior, middle, and posterior disc heights divided by the sum of the depths of the adjacent cranial and caudal vertebral bodies. For longitudinal analysis, DHI at each follow up was expressed relative to the corresponding baseline value for each animal.

An additional eight rats were used for radiographic mechanical characterization. For each prescribed displacement setting, paired radiographs were obtained from the same animal under unloaded and peak displacement conditions. Radiographic disc deformation was calculated as the percentage reduction in disc height relative to the corresponding unloaded measurement: Radiographic disc height deformation (%) = [(unloaded disc height − loaded disc height)/unloaded disc height] × 100%.

To determine whether compression produced motion segment wedging or sagittal plane A–P translation, paired sagittal radiographs from the eight characterization animals were analyzed at 0.0, 0.4, 0.8, and 1.2 mm of prescribed K‐wire displacement. Four ordered landmarks were placed from image left to right along the inferior endplate of the cranial vertebra (A–D), and four points were placed along the superior endplate of the caudal vertebra (E–H; Figure 2D). Each landmark set was fitted with a line using total least squares. The fitted axis of the superior endplate of the caudal vertebra served as the local reference axis. Wedge angle was calculated as the angle of the caudal superior endplate minus the angle of the cranial inferior endplate and was expressed between −90° and 90°. Because image y coordinates increased downward, a positive value indicated a disc space that was narrower on the image left and opened toward the image right. The signed change in wedge angle at each loaded state was calculated relative to the corresponding unloaded measurement.

**FIGURE 2:**
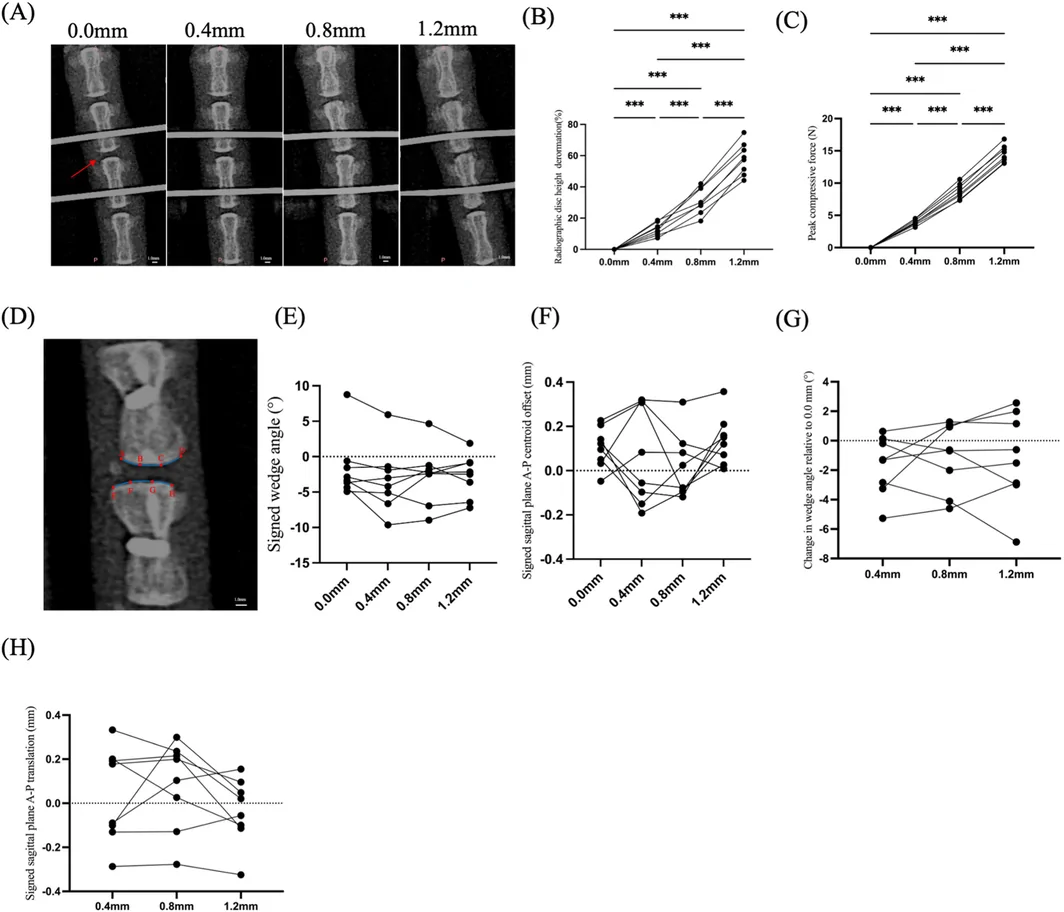
Mechanical characterization of motion segment deformation during graded prescribed K‐wire displacement. (A) Representative anteroposterior radiographs from the same animal obtained under the unloaded condition (0.0 mm) and at peak K‐wire displacements of 0.4, 0.8, and 1.2 mm. The red arrow indicates the target Co8–Co9 disc. (B) Radiographic disc height deformation derived from paired unloaded and peak displacement radiographs. (C) Peak compressive force measured at each displacement. (D) Four landmarks along the inferior endplate of the cranial vertebra (A–D) and four along the superior endplate of the caudal vertebra (E–H). (E) Signed wedge angle and (F) signed sagittal plane A–P centroid offset measured under the unloaded and three loaded conditions. (G) Change in wedge angle and (H) sagittal plane A–P translation, each calculated by subtracting the corresponding unloaded measurement for each animal. Positive values indicate opening toward image right and movement of the upper centroid toward image right, respectively. Lines connect repeated measurements from the same animal (*n* = 8). Scale bars, 1.0 mm; ***p < 0.001.

Sagittal plane A–P translation was determined from the centroids of the four cranial inferior endplate landmarks and the four caudal superior endplate landmarks (Figure 2D). The vector extending from the caudal centroid to the cranial centroid was projected onto a unit vector directed from image left to right along the fitted caudal superior endplate axis. The projected value in the unloaded image was subtracted from that in each loaded image and converted to millimeters using a scale of 0.125 mm/pixel. Positive values indicated movement of the cranial inferior endplate centroid toward image right relative to the caudal superior endplate; negative values indicated movement toward image left. The method was adapted from the centroid projection framework described by Meadows et al. [22] Because complete disc segmentations and whole vertebral bone point sets were unavailable, the fitted axis of the superior endplate of the caudal vertebra was used in place of the disc principal axis, and wedge angulation was derived from the relative orientation of the two fitted endplate lines rather than rigid body registration of the vertebrae. The resulting translation is a two‐dimensional kinematic measure in the sagittal image plane and should not be interpreted as shear force, shear stress, or three‐dimensional shear strain. The wedge angles and sagittal plane A–P centroid offsets for each loading condition are shown in Figure 2E,F, respectively; their signed changes relative to the unloaded condition are shown in Figure 2G,H. The same anatomical landmarks and imaging orientation were used for all images. Two investigators blinded to group allocation performed the measurements independently, and their values were averaged for analysis.

### 2.6 Magnetic Resonance Imaging (MRI)

MRI was performed in all animals at the end of the 4‐week loading period according to the predefined experimental protocol. Imaging was performed using a 3.0‐T magnetic resonance imaging system. Coronal spin‐echo T2‐weighted images were acquired using the following parameters: repetition time/echo time (TR/TE), 3422/45 ms; slice thickness, 1.0 mm; interslice gap, 0.2 mm; matrix size, 288 × 128; and field of view, 160 × 160 mm. T2‐weighted images were independently evaluated by two investigators blinded to group allocation using the Pfirrmann grading system (grades I–V). In cases of disagreement, a consensus score was assigned following joint review.

### 2.7 Histological Analysis and Cartilaginous Endplate Morphometry

After completion of imaging assessments, animals were euthanized by anesthetic overdose. The Co8–Co9 motion segment was harvested and surrounding soft tissues were carefully removed. Specimens were fixed in 4% paraformaldehyde for 24 h and decalcified in 10% ethylenediaminetetraacetic acid (EDTA) for 30 days. Following dehydration and paraffin embedding, sagittal sections (5 μm thickness) were prepared.

For hematoxylin and eosin (HE) staining, sections were deparaffinized, rehydrated through graded ethanol solutions, stained with hematoxylin, differentiated, blued, and counterstained with eosin. After dehydration and clearing, sections were mounted with neutral resin. For safranin O‐fast green staining, deparaffinized and rehydrated sections were stained with fast green, differentiated in acid alcohol, counterstained with safranin O, dehydrated, cleared, and mounted. Scale bars were included in all representative histological images.

Histological degeneration was evaluated according to the Orthopedic Research Society (ORS) rat intervertebral disc histopathology scoring system [23]. Eight features were assessed: NP shape, NP area, NP cell number, NP cell morphology, NP–AF border appearance, AF lamellar organization, AF tears or fissures, and endplate abnormalities. Each feature was scored from 0 to 2, resulting in a total score of 0–16, with higher scores indicating more severe degeneration. The endplate item integrates disruptions or microfractures and osteophyte or ossification: score 0 indicates a continuous endplate without osteophyte or ossification; score 1 indicates minimal disruption or mild osteophyte or ossification involving less than one third of the endplate; and score 2 indicates disruption involving at least one third or substantial osteophyte or ossification involving at least one third. The inferior endplate of the cranial vertebra and the superior endplate of the caudal vertebra were scored separately according to the ORS endplate criteria. For the animal level analysis, the higher of the two scores was recorded as the maximum endplate abnormality score. An animal was classified as having an endplate abnormality if either endplate received a score greater than 0. Score distributions and the number of affected animals were reported descriptively for each group.

Cartilaginous endplate morphometry was performed on calibrated digital images of hematoxylin and eosin stained sections taken through the central coronal plane of the Co8–Co9 disc. The cartilaginous endplate was identified as the cartilage layer directly facing the disc and was distinguished from the more deeply located vertebral growth plate using published descriptions of rat endplate anatomy [24]. The inferior cartilaginous endplate of the cranial vertebra and the superior cartilaginous endplate of the caudal vertebra were measured separately and then averaged to obtain one animal level mean for each morphometric outcome. For each endplate, four ordered landmarks were placed along the identifiable disc facing contour, and endplate contour length was calculated as the sum of the three Euclidean distances between consecutive landmarks. Cartilaginous endplate thickness was measured perpendicular to the disc facing and vertebral facing boundaries at three locations on each endplate: the central region and the two peripheral regions. The six measurements from the cranial and caudal cartilaginous endplates were averaged to obtain one animal level mean thickness. This was a study specific morphometric adaptation; Li et al. [24] used toluidine blue sections for thickness measurement, whereas hematoxylin and eosin sections were used in the present study to permit morphometry and pathological assessment on the same section. Two researchers blinded to group allocation independently performed the histological grading, and disagreements were resolved by consensus.

### 2.8 Immunohistochemistry

Sections were placed in citrate buffer (pH 6.0) and subjected to high‐pressure heat‐induced antigen retrieval for 2 min, then cooled to room temperature. Endogenous peroxidase was blocked with 3% H_2_O_2_ for 15 min, followed by three washes with PBS. Sections were then blocked with 5% normal goat serum for 30 min. Primary antibodies against collagen I (COL‐I) (Abclonal, 1:100), collagen II (COL‐II) (Abclonal, 1:2000), aggrecan (ACAN) (Proteintech, 1:500), and matrix metalloproteinase 13 (MMP‐13) (Servicebio, 1:100) were applied overnight at 4°C. After three PBS washes, an HRP‐conjugated goat anti‐rabbit (Servicebio, 1:500) secondary antibody was applied for 60 min. Immunoreactivity was visualized using DAB chromogen, and the reaction was terminated with distilled water. Sections were counterstained with hematoxylin, dehydrated, cleared, and mounted.

Immunohistochemical quantification was performed separately in the NP and AF. For each animal, one representative section was selected for analysis. Within each region, three nonoverlapping microscopic fields were randomly selected at ×200 magnification and analyzed under identical imaging conditions. The percentage of positively stained area (%Area) was quantified using ImageJ software. The mean value obtained from the three fields was used as the final measurement for each animal. All image analyses were performed by investigators blinded to group allocation.

### 2.9 Statistical Analysis

Continuous data were expressed as mean ± standard deviation (SD). Statistical analyses were performed using GraphPad Prism version 10.5.0. Data normality was assessed using the Shapiro–Wilk test, and homogeneity of variance was evaluated using Levene's test.

Longitudinal DHI measurements were analyzed using two‐way repeated measures analysis of variance (ANOVA). Peak compressive force and radiographic disc height deformation across the three displacement settings were analyzed using one‐way repeated measures ANOVA. Endpoint DHI at week 4, normalized to the sham group, was analyzed using one‐way ANOVA followed by Tukey multiple comparisons. Baseline‐unadjusted wedge angles and sagittal plane A–P centroid offsets at 0.0, 0.4, 0.8, and 1.2 mm prescribed K‐wire displacement were analyzed using the Friedman test. Because neither omnibus test was statistically significant, no post hoc comparisons were performed. Signed changes relative to 0.0 mm were presented descriptively. Animal level mean endplate contour length, mean cartilaginous endplate thickness, and immunohistochemical measurements were analyzed using one‐way ANOVA followed by Tukey multiple comparisons. ORS histopathology scores, maximum endplate abnormality scores, and Pfirrmann grades were analyzed using the Kruskal–Wallis test followed by Dunn multiple comparisons. Endplate abnormality frequency and the distribution of scores 0, 1, and 2 were reported descriptively as animal counts. Normally distributed continuous data are presented as mean ± SD, whereas ordinal data are presented as median (IQR). All statistical tests were two‐tailed, and *p* < 0.05 was considered statistically significant.

## 3 Results

### 3.1 Mechanical Characterization and Motion Segment Deformation

In the separate characterization cohort, radiographic disc height deformation and peak compressive force increased with prescribed K‐wire displacement (*p* < 0.001). Paired radiographs from the same eight animals showed that displacements of 0.4, 0.8, and 1.2 mm produced mean disc height deformations of 13.00% ± 4.05%, 31.11% ± 8.38%, and 58.08% ± 10.27%, respectively (Figure 2A,B). At the same displacement settings, the mean peak compressive forces were 3.94 ± 0.46, 8.69 ± 1.15, and 14.54 ± 1.31 N, respectively (Figure 2C).

Wedge angles and sagittal plane A–P centroid offsets measured under the unloaded condition and at 0.4, 0.8, and 1.2 mm prescribed K‐wire displacement are shown in Figure 2E,F, respectively. Neither measure differed significantly among the four displacement conditions (*p* > 0.05). Changes in wedge angle and sagittal plane A–P translation relative to each animal's unloaded measurement are shown in Figure 2G,H, respectively. These changes occurred in both directions and did not differ significantly among the three loaded displacement conditions (*p* > 0.05).

### 3.2 Imaging and Histological Outcomes After Four Weeks

After 4 weeks, the low compression group had a radiographic and MRI appearance similar to the sham group. The moderate compression group maintained MRI signal and showed a higher endpoint DHI than sham, whereas the high compression group showed disc space narrowing, loss of T2 signal, and structural disruption (Figure 3A,D). Pfirrmann grades did not differ significantly among the sham, low compression, and moderate compression groups. The high compression group had higher grades than the other groups (adjusted *p* < 0.05; Figure 3E).

**FIGURE 3:**
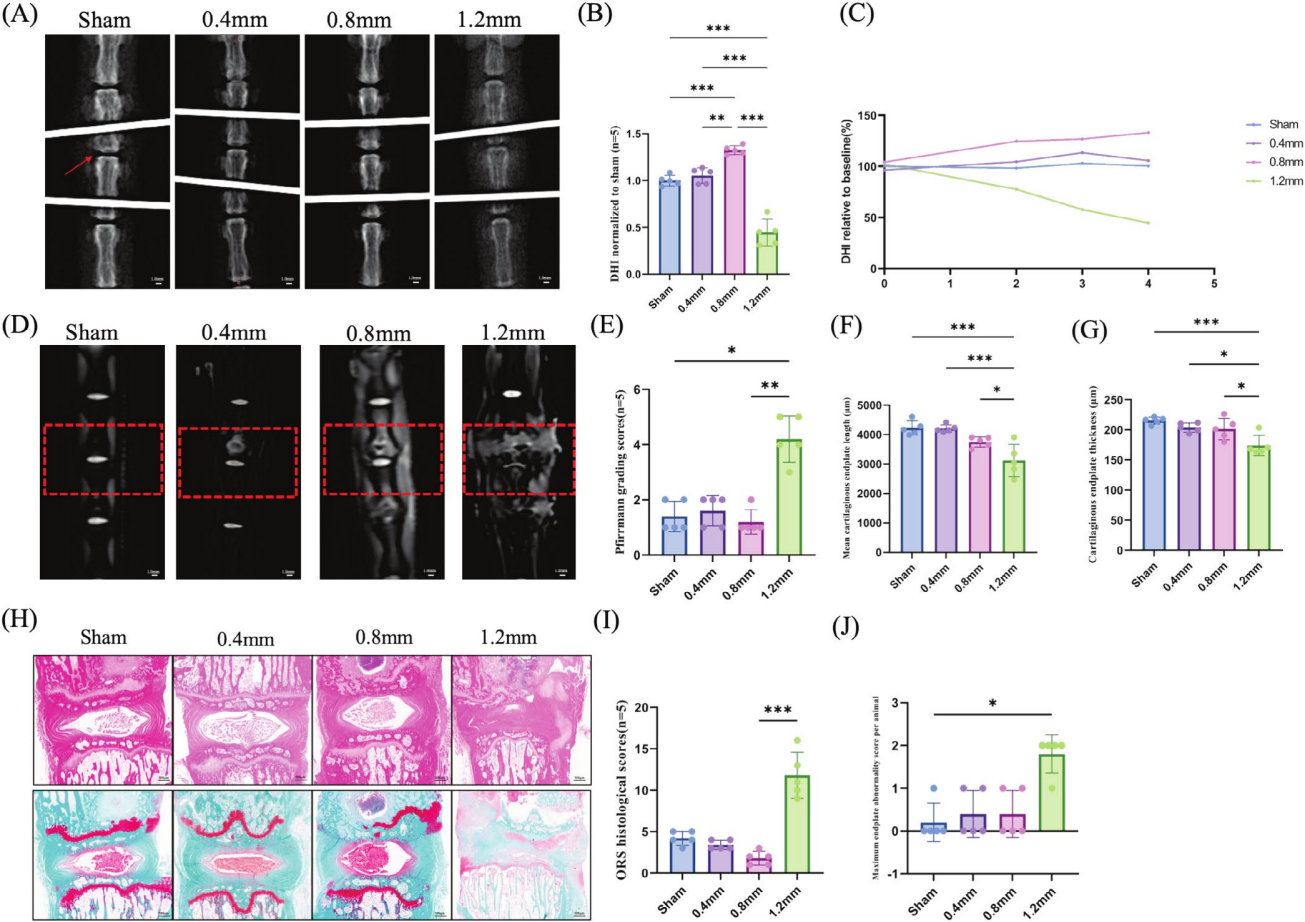
Imaging, histological, and cartilaginous endplate outcomes after four weeks of graded cyclic compression. Groups are sham, low compression (0.4 mm), moderate compression (0.8 mm), and high compression (1.2 mm). (A) Representative sagittal radiographs; the red arrow marks an endplate defect adjacent to the target Co8–Co9 disc. (B) Endpoint disc height index (DHI) normalized to the sham group. (C) Longitudinal DHI expressed relative to each animal's baseline. (D) Representative coronal T2 weighted magnetic resonance images; red dashed boxes identify Co8–Co9. (E) Pfirrmann grades. (F) Mean endplate contour length after averaging the cranial and caudal measurements within each animal. (G) Mean cartilaginous endplate thickness after averaging the cranial and caudal measurements within each animal. (H) Representative midsagittal hematoxylin and eosin stained sections (top row) and safranin O and fast green stained sections (bottom row). (I) Total ORS histopathology score. (J) Animal level maximum endplate abnormality score, defined as the larger score from the cranial and caudal endplates. Scale bars are shown in each image; *p < 0.05, **p < 0.01, ***p < 0.001.

Longitudinal DHI% differed among the displacement groups. Values remained relatively stable in the sham and low compression groups, increased gradually in the moderate compression group, and decreased in the high compression group. At week 4, the moderate compression group had a higher DHI than the sham group (*p* < 0.001), while the high compression group had a lower DHI than both the sham and moderate compression groups (*p* < 0.001) (Figure 3B). The longitudinal trajectories are shown in Figure 3C.

Histological examination revealed loading‐dependent changes in disc morphology among the experimental groups. The sham and low compression groups retained a defined nucleus pulposus and organized annulus fibrosus. The moderate compression group also showed a preserved nucleus pulposus and orderly annular lamellae. In contrast, the high compression group demonstrated pronounced degenerative changes, including loss of NP tissue, disruption of NP architecture, and disorganization of the AF lamellar structure (Figure 3H). ORS histopathology scores differed significantly among the four groups. Post hoc analysis identified a significant difference only between the moderate and high compression groups (adjusted *p* < 0.001); all other pairwise comparisons were not significant (Figure 3I).

At 4 weeks, after averaging the cranial and caudal measurements within each animal, mean endplate contour length was lower in the high compression group than in the sham and low compression groups (*p* < 0.05; Figure 3F). Mean cartilaginous endplate thickness was also lower in the high compression group than in the sham group (*p* < 0.001) and the low and moderate compression groups (*p* < 0.05; Figure 3G). Endplate defects were observed in one of five sham animals, two of five low compression animals, two of five moderate compression animals, and all five high compression animals. Ossification was observed in two high compression animals but not in the other groups, and one defect in the high compression animal had a defect involving the central cartilaginous endplate. The animal level maximum endplate abnormality score was higher in the high compression group than in the sham group (adjusted *p* < 0.05; Figure 3J). Given the small group size, lesion frequencies were summarized descriptively.

### 3.3 Immunohistochemistry Results

Representative immunohistochemical staining images of COL‐I, COL‐II, ACAN, and MMP‐13 in the NP and AF are shown in Figure 4A. Among the analyzable NP groups, the low compression group had a higher COL‐II‐positive area than the sham group (*p* < 0.05). The moderate compression group had a higher COL‐II‐positive area than the sham and low compression groups (*p* < 0.001) and a higher ACAN‐positive area than the sham (*p* < 0.01) and low compression groups (*p* < 0.001). NP MMP‐13 staining was also higher in the moderate compression group than in the sham and low compression groups (*p* < 0.001; Figure 4B).

**FIGURE 4:**
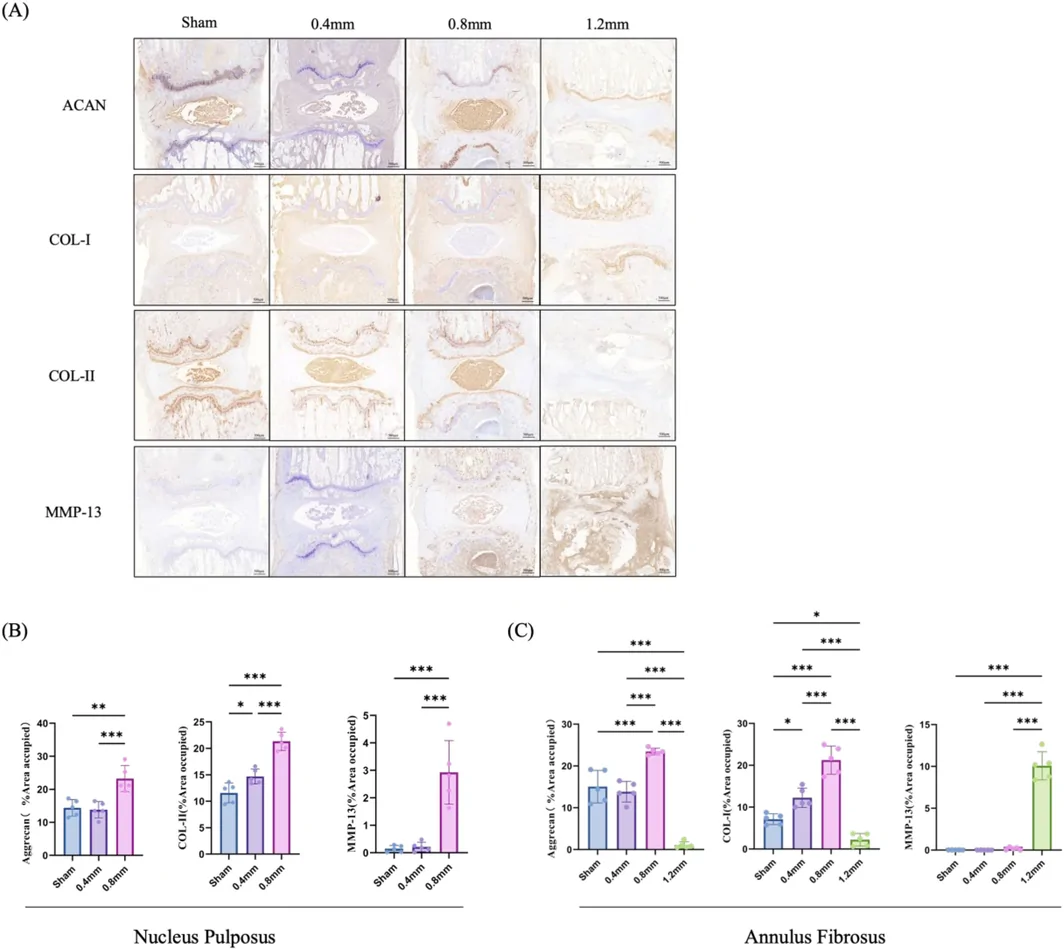
Immunohistochemical staining of matrix‐associated and catabolic markers after 4 weeks of graded cyclic compression. (A) Representative sagittal staining for aggrecan (ACAN), collagen I (COL‐I), collagen II (COL‐II), and MMP‐13 in the sham, low (0.4 mm), moderate (0.8 mm), and high (1.2 mm) compression groups. Brown signal indicates positive staining. Scale bars, 500 μm. (B) Percentage positive area for ACAN, COL‐II, and MMP‐13 in the analyzable nucleus pulposus groups (sham, low compression, and moderate compression; *n* = 5 per group). The high compression group was excluded from NP quantification because identifiable NP tissue was not retained. (C) Percentage positive area for COL‐I, ACAN, and MMP‐13 in the annulus fibrosus. Data are presented as mean ± SD with individual values (*n* = 5 per group); *p < 0.05, **p < 0.01, ***p < 0.001.

In the AF, the low compression group had a higher COL‐I‐positive area than the sham group (*p* < 0.05). The moderate compression group had higher COL‐I‐ and ACAN‐positive areas than the sham, low compression, and high compression groups (*p* < 0.001). The high compression group had lower COL‐I‐ and ACAN‐positive areas than the sham and low compression groups, whereas MMP‐13 staining was higher than in all three other groups (Figure 4C).

## 4 Discussion

The present study established a motor‐driven five‐channel system for cyclic compression of the rat caudal intervertebral disc and used it to determine how loading amplitude influenced both the mechanical response and the tissue responses. Increasing prescribed K‐wire displacement produced ordered increases in initial peak compressive force at the fixation assembly and radiographic disc height deformation, but the biological response was not a simple linear escalation. Low and moderate compression retained overall disc architecture while showing evidence of matrix remodeling, whereas high compression was associated with disc narrowing, loss of nucleus pulposus structure, annular catabolic remodeling, and more frequent and severe cartilaginous endplate abnormalities. Neither wedge angle nor sagittal plane A–P centroid offset differed significantly among the four displacement states. Taken together, these findings indicate that the system delivered predominantly axial compression at the group level and generated amplitude‐dependent tissue responses, with these responses ranging from structurally compensated remodeling to an endplate‐associated degenerative phenotype.

A possible explanation for this graded response is that cyclic compression changes fluid movement, cell deformation, and local matrix stress throughout the motion segment, with the balance between adaptation and injury depending on mechanical dose [4, 6, 25]. At lower amplitude, these stimuli may promote matrix turnover before a change becomes detectable by disc height, magnetic resonance imaging, or routine histology. The greater matrix marker positive areas at low and moderate compression are compatible with such remodeling, but they should not be interpreted as direct measurements of matrix synthesis. In particular, the concurrent increase in nucleus pulposus MMP‐13 at moderate compression suggests that matrix accumulation and degradation may coexist in a compensated state. At high compression, the large axial deformation may exceed the capacity of the nucleus pulposus, annulus fibrosus, and cartilaginous endplates to distribute load. Loss of nucleus pressurization could then shift stress toward the annulus and endplate interfaces, reinforcing structural disruption and catabolic remodeling. The accompanying endplate defects, reduced cartilaginous endplate thickness, shortened visible endplate contours, and occasional ossification are consistent with this whole motion segment interpretation. Experimental cyclic compression has been shown to initiate and accumulate microstructural damage within the cartilaginous endplate [26], while human degeneration involves coordinated changes across the disc, cartilaginous endplate, and vertebral bone [27, 28].

The findings are consistent with the broader loading literature while also illustrating why comparisons based only on device input are incomplete. In vivo rat tail studies have shown that disc gene expression, composition, and mechanical behavior depend on loading magnitude, frequency, and exposure duration [13, 15, 16, 20]. MacLean et al. found magnitude and frequency dependent anabolic and catabolic transcription, whereas Wuertz et al. reported predominantly anabolic remodeling with shorter dynamic loading and mild degenerative changes after longer exposure [15, 20]. Iatridis et al. further showed that excessive dynamic compression increased MMP‐associated aggrecan degradation before major structural disruption became evident [16]. More recently, whole organ rat tail experiments demonstrated magnitude dependent inflammatory and matrix loss responses to cyclic compression [25]. In contrast to regimens that produced limited structural injury, the highest setting in the present study produced marked disc and endplate damage. This difference is plausible because prescribed displacement, transmitted force, tissue stress, frequency, waveform, daily duration, and total exposure are not interchangeable components of mechanical dose.

The endplate findings refine the degenerative phenotype represented by this model. In humans, structural endplate defects are associated with lower disc signal and height, disc displacement, Modic changes, and greater disability, and cartilaginous endplate avulsion has been associated with adjacent endplate defects and Modic changes [29, 30]. Human tissue studies also describe cartilaginous endplate thinning, fissuring, matrix disorganization, and calcification as degeneration advances [27, 28]. The high compression group reproduced selected elements of this constellation, including disc narrowing and T2 signal loss, nucleus pulposus disruption, annular catabolic remodeling, reduced cartilaginous endplate dimensions, defects, and occasional ossification. These findings support describing the model as a mechanically induced, endplate associated degenerative phenotype. The biological plausibility of disc and endplate crosstalk is reinforced by a rat lumbar endplate microfracture model in which endplate injury produced disc height loss, reduced T2 signal, vertebral remodeling, and Modic‐like marrow changes [31]. However, the present study did not assess marrow sensitive MRI, microcomputed tomography, pain behavior, or nucleus pulposus herniation into vertebral bone. The abnormalities should therefore not be presented as Schmorl nodes, Modic changes, a pain phenotype, or a complete model of spontaneous human degeneration. They reproduce selected structural and matrix features of an endplate associated human degenerative pathway.

The significance of this work therefore lies in the experimental framework rather than in claiming that dynamic rat tail compression is itself new. Earlier external fixation and pneumatic systems established the feasibility of controlled in vivo loading and simultaneous testing of multiple animals [15, 20, 32]. Recent model evaluations emphasize that translational value depends on matching a clearly defined experimental phenotype to the research question and on acknowledging differences in species scale, metabolism, and repair capacity [33, 34]. The present system combines a simple, low‐cost, motor‐driven architecture with five parallel loading channels and directly relates three prescribed K‐wire displacement settings to initial force at the fixation assembly, radiographic disc height deformation, wedge angulation, sagittal plane A–P translation, and subsequent tissue outcomes. Interpreting the device setting alongside these measured motion segment responses provides a clearer description of mechanical exposure than the nominal actuator input alone. The system also preserves an intact annulus, unlike puncture models that initiate degeneration through immediate tissue disruption and provides more than one severity state for mechanistic or intervention studies [17]. Use of the standardized ORS rat histopathology framework further supports comparison with other rat disc models [23]. For mechanobiology researchers, the model enables loading amplitude to be varied while the resulting compressive force, axial disc deformation, and tissue response are evaluated within the same experimental framework. For endplate and imaging researchers, it provides a platform for investigating how endplate abnormalities develop in relation to disc degeneration under cyclic axial compression. For preclinical therapy and tissue engineering studies, the system can be used to compare preventive, early intervention, and treatment strategies across defined loading severities; previous work showing that restoration of mechanical loading influenced engineered disc integration illustrates the importance of a controllable mechanical environment [35]. The lower loading settings may be useful for studying early mechanotransduction and potentially reversible remodeling, whereas the high setting permits investigation of structural damage involving both the disc and cartilaginous endplates. By producing responses that range from compensated remodeling to structural degeneration, the model may help determine whether an intervention alters the transition from adaptation to tissue damage rather than merely improving a single advanced endpoint.

Several limitations temper these conclusions. Each 4‐week group contained five animals, and the mechanical characterization was performed in a separate cohort of eight animals. The measured force and radiographic deformation therefore define the initial output associated with each prescribed displacement but do not show that force, pressure, or percentage deformation remained constant as the disc changed over time. Force was measured through the K‐wires and fixation assembly rather than within the disc. The protocol controlled displacement and did not provide closed loop control of force or intradiscal pressure. Animals in the loading groups were placed in the holder during daily compression, whereas sham animals remained active in their cages; systemic effects of restraint cannot therefore be separated completely from the effects of cyclic displacement. Only male rats were studied, although sex influences disc structure and responses to injury in rat models [18, 19]. Rat caudal discs also differ from human lumbar discs in anatomy, cell phenotype, posture, and loading environment. In particular, the rat cartilaginous endplate is directly connected to the growth plate, and species differences in scale and matrix turnover limit direct translation of both the loading dose and repair response [24, 33]. Only one frequency, one daily duration, and one endpoint were evaluated. Sagittal radiographs cannot capture out of plane motion, and two dimensional histological sections cannot define total endplate defect volume. Finally, immunohistochemistry measured marker positive area rather than synthesis rate, enzyme activity, cleavage products, or collagen organization. Future studies should combine serial force recording with longitudinal endplate sensitive imaging, three dimensional lesion assessment, pain related outcomes, both sexes, and restraint matched controls to determine the temporal sequence and translational relevance of the observed phenotype.

In conclusion, this study establishes a mechanically characterized framework for examining loading amplitude effects in the rat caudal intervertebral disc. Its advance is not the use of cyclic compression alone, but the integration of five channel motor‐driven loading with direct characterization of prescribed K‐wire displacement, peak force at the fixation assembly, radiographic disc height deformation, wedge angulation, sagittal plane A–P translation, and multiscale tissue outcomes. Within the resolution of two‐dimensional radiography, axial disc height reduction was the predominant group level deformation. The biological findings identify structurally preserved remodeling at lower amplitudes and a high amplitude phenotype characterized by disc collapse, matrix disruption, catabolic activity, and frequent endplate defects with occasional ossification. This tunable distinction between compensation and injury provides the clearest rationale for the model and a practical basis for future studies of mechanical thresholds, interactions between the disc and endplate, and load‐targeted interventions.

## Author Contributions

**Kaixuan Jiang:** conceptualization, investigation, writing – original draft. **Wei Cui:** formal analysis, writing – original draft, methodology, software. **Yin Zhuang:** visualization. **Daoguang Tian:** data curation, validation. **Wei Peng:** writing – review and editing, project administration. **Shujun Zhang:** writing – review and editing, supervision, resources, funding acquisition.

## Funding

This work was supported by Wuxi Municipal Health Commission (T202407).

## Conflicts of Interest

The authors declare no conflicts of interest.

## Supporting information


**Video S1:** Operation of the five‐channel motor‐driven compression system for rat caudal intervertebral disc loading.

## Data Availability

The data that support the findings of this study are available from the corresponding author upon reasonable request.
